# Optimization strategy for the early timing of bronchoalveolar lavage treatment for children with severe mycoplasma pneumoniae pneumonia

**DOI:** 10.1186/s12879-023-08619-9

**Published:** 2023-10-05

**Authors:** Xiangtao Wu, Weihong Lu, Tuanjie Wang, Aiju Xiao, Xixia Guo, Yali Xu, Shujun Li, Xue Liu, Hanshi Zeng, Shaoru He, Xingliang Zhang

**Affiliations:** 1 Department of Pediatrics, the First Affiliated Hospital of Xinxiang Medical University, Weihui, 453100 China; 2 The Second School of Clinical Medicine, Southern Medical University, Guangzhou, 510260 China; 3 Department of Neonatology, Guangdong Provincial People′s Hospital (Guangdong Academy of Medical Sciences), Southern Medical University, Guangzhou, 510080 China; 4 Department of Respiratory Medicine, Institute of Pediatrics, Shenzhen Children’s Hospital, Shenzhen, 518038 China

**Keywords:** Severe mycoplasma pneumoniae pneumonia, Nomogram model, Therapeutic bronchoalveolar lavage, Kaplan–Meier analysis, Children

## Abstract

**Background:**

Early evaluation of severe mycoplasma pneumoniae pneumonia (SMPP) and the prompt utilization of fiberoptic bronchoscopic manipulation can effectively alleviate complications and restrict the progression of sequelae. This study aim to establish a nomogram forecasting model for SMPP in children and explore an optimal early therapeutic bronchoalveolar lavage (TBAL) treatment strategy.

**Methods:**

This retrospective study included children with mycoplasma pneumoniae pneumonia (MPP) from January 2019 to December 2021. Multivariate logistic regression analysis was used to screen independent risk factors for SMPP and establish a nomogram model. The bootstrap method was employed and a receiver operator characteristic (ROC) curve was drawn to evaluate the accuracy and robustness of the model. Kaplan–Meier analysis was used to assess the effect of lavage and hospitalization times.

**Results:**

A total of 244 cases were enrolled in the study, among whom 68 with SMPP and 176 with non-SMPP (NSMPP). A prediction model with five independent risk factors: left upper lobe computed tomography (CT) score, sequential organ failure assessment (SOFA) score, acute physiology and chronic health assessment (APACHE) II score, bronchitis score (BS), and c-reactive protein (CRP) was established based on the multivariate logistic regression analysis. The ROC curve of the prediction model showed the area under ROC curve (AUC) was 0.985 (95% confidence interval (CI) 0.972–0.997). The Hosmer–Lemeshow goodness-of-fit test results showed that the nomogram model predicted the risk of SMPP well (χ2 = 2.127, *P *= 0.977). The log-rank result suggested that an early BAL treatment could shorten MPP hospitalization time (*P* = 0.0057).

**Conclusion:**

This nomogram model, based on the left upper lobe CT score, SOFA score, APACHE II score, BS, and CRP level, represents a valuable tool to predict the risk of SMPP in children and optimize the timing of TBAL.

## Introduction

Mycoplasma pneumoniae pneumonia (MPP) is a common cause of community-acquired pneumonia in children, especially school-age children [1]. The incidence of severe mycoplasma pneumoniae pneumonia (SMPP) in Kutty’s study was approximately 12% [2] while that in Izumikawa’s study was approximately 0.5–2% [3]. SMPP can cause various pulmonary and extrapulmonary complications, including encephalitis, myocarditis, and hemolytic anemia [4, 5]. The definition of SMPP mainly relying on clinical and radiological manifestations as well as microbiological and serological tests. However, there is currently no consensus on the definition and diagnosis of SMPP [6]. Due to the increase in drug resistance and irrational use of antibiotics, some patients even develop severe complications such as acute respiratory distress syndrome, which requires extracorporeal membrane lung oxygenation treatment [7]. Hence, it is imperative to ascertain a standardized and objective approach for evaluating the severity of MPP and forecasting SMPP.

Bronchoalveolar lavage (BAL) has been used to treat various lung diseases, including cystic fibrosis, pulmonary alveolar proteinosis [8]. Few studies have investigated the effectiveness of BAL treatment for children with SMPP. The early therapeutic BAL(TBAL)treatment (receiving BAL within 1 day of admission) was considered to be beneficial for patients with severe pneumonia by reducing the hospitalization time [9]. The lack of standardized criteria and definitions for SMPP and the potential risks and complications of BAL make it difficult to compare the results across studies, which may outweigh the benefits in some cases [10]. Additionally, only some studies have quantitatively assessed bronchial inflammation by bronchoscopy, and the timing of TBALfor MPP needs to be clarified.

Nomogram can transform complex regression equations into visual graph, making prediction model results more readable and convenient for patient assessment [11]. Therefore, in this study, we collected 244 patients for retrospectively analyzing independent risk factors for SMPP, and then established a nomogram-based prediction model to explore the best strategy for early BAL treatment for children with SMPP.

## Research method

### Object

The subjects included 684 children with MPP admitted to the Department of Pediatrics of the First Affiliated Hospital of Xinxiang Medical University from January 2019 to December 2021, among whom 244 patients completed TBAL treatment. The inclusion criteria for non-SMPP(NSMPP) were as follows: Meet the diagnostic criteria for pneumonia [11]; MP-DNA positive by multiple polymerase chain reaction (PCR) of BAL fluid and indications for BAL include radiologically confirmed large pulmonary lesions, consolidation, atelectasis and poor response to macrolide treatment. The parents and relatives need to consent to the bronchoalveolar lavage and signed the informed consent form. The exclusion criteria were as follows: the attending physician considered that the cause of the disease was other pathogens; patients with chronic respiratory diseases (e.g., bronchiectasis, asthma, congenital heart disease, pulmonary hypertension, active pulmonary tuberculosis), congenital inherited metabolic diseases, malignant tumors; patients with cardiac arrest, organ transplantation, or surgery; patients with a hospital-acquired infection, immunodeficiency, or use of immunosuppressive drugs; incomplete BAL treatment; and incomplete information. The diagnostic criteria for SMPP were as follows [12]: NSMPP patients had at least three of the following five items: dyspnea and chest retraction (less than 1-year-old: RR ≥ 50 beats/min, HR ≥ 150 beats/min; 1–5 years old: RR ≥ 40 beats/min, HR ≥ 140 beats/min; over 5 years old: RR ≥ 30 beats/min, HR ≥ 120 beats/min); Oxygenation index < 250 mmHg; Hypercapnia; Altered consciousness; Elevated blood urea nitrogen; and Hypotension requiring fluid resuscitation. The Ethics Committee of our hospital approved this study protocol (EC-022-044) and waived the requirement for informed consent due to retrospective nature of the study.

### Study design

Grouping: All patients were divided into SMPP and NSMPP groups [12] according to the severity of the disease. In addition, according to the fiberoptic bronchoscopy (FOB) time after admission, the patients were divided into early TBAL group (receiving TBAL within 24 h of admission) and late TBAL group(receiving TBAL after 24 h of admission). Demographic characteristics(e.g., sex, age, length of hospital stay, time of onset before ICU admission), clinical manifestations(e.g., fever, cough, wheezing), laboratory tests(e.g., MP-DNA, procalcitonin (PCT), interleukin-6 (IL-6), immunoglobulin, lymphocyte subsets), computed tomography (CT) score, sequential organ failure assessment (SOFA) score, Acute Physiology and Chronic Health Assessment (APACHE) II scores before BAL, and bronchitis score (BS) by re-analyzed of FOB were collected.

### FOB and BAL

Lavage solution was instilled 3–5 times at 1 ml/kg/time. A solution of 0.9% sodium chloride was utilized for the BAL procedure and heated to a temperature of 37℃. The saline solution was recovered under a negative pressure of 6.65–13.3 kPa (50–100 mmHg); the volume of BAL fluid recovery was 40% and more. The combination of Midazolam(0.1-0.3 mg/kg per time, 1ml:5 mg, Jiangsu Enhua Pharmaceutical Co., LTD., H20143222) and propofol (2.5 mg/kg, 20ml:200 mg, Fresenius Kabi AB, J20130013H20143222) was utilized in all procedures to achieve moderate sedation. Additionally, lidocaine hydrochloride(1-2ml per time, 5ml:100 mg, Shanghai Zhaohui Pharmaceutical Co., LTD., H31021071) was applied at the nasal and alveolar lavage site to minimize cough reflex and respiratory spasm. Satisfactory sedation was achieved after 1–2 doses of the Midazolam and propofol combination, and it lasted for 20 min until the completion of the procedure. In addition to measuring the heart rate, respiration, and blood oxygen saturation using the ECG monitor, sufficient oxygen supply was also ensured.

### Bronchitis scoring

The bronchoscopy video data were reviewed and scored by several experienced bronchoscopists unaware of the patient’s clinical history. The scoring sites were in the trachea, right main bronchus, right upper lobe, right bronchus intermedius, right middle lobe, right lower lobe, left main bronchus, left upper lobe (including lingula), and left lower lobe, totaling nine sites. Each site was scored for the following six bronchoscopic visual features: amount and color of secretions, presence or absence of mucosal edema, elevation, erythema, and pallor. The volume score is a measure of the amount of secretion in the bronchial lumen. It ranges from 1 to 6, with higher scores indicating a greater presence of secretions. The scoring system for secretions is as follows:Score 1: No secretions are observed in any sites. Score 2: Bubbly secretions are present in less than 5 sites, but do not fill the bronchial lumen.Score 3: Bubbly or viscous secretions are present in more than 5 sites, and fill less than 1/3 of the bronchial lumen.Score 4: Viscous secretions are present in any site, and fill more than 1/3 of the bronchial lumen, or less than 1/2 of the sites have viscous secretions.Score 5: More than 1/2 of the sites have viscous secretions, or less than 1/2 of the sites have bronchial lumens blocked by secretions.Score 6: More than half of the bronchial lumens are blocked by secretions. The color of the secretions was scored according to the BronkoTest® sputum color chart, with a color score ranging from 0 to 8. The first round of scoring was conducted as follows: mucosal edema, eminence, erythema, and pallor features were scored from 0 to 2 according to severity level (0, none; 1, mild; 2, moderate to severe). The second round correction scoring was conducted as follows: For each site’s mucosal appearance, a composite score from 0 to 3 was given based on the number of affected sites (0, none; 1, < 50% of the nine scoring sites received 1 point; 2, > 50% of the nine sites received 1 point; 3, > 50% of the nine sites scored > 2 points) [13]. For cases with large differences in scores between the two physicians, the case was re-scored by both physicians simultaneously to ensure the reliability and consistency of the bronchitis score results.

### CT scoring

CT scoring [14] was first assigned according to the extent of ground-glass opacity changes involving lung lobes (each of the five lung lobes could have a maximum score of 5), with scores defined as follows: 0 for no involvement; 1 for < 5% involvement; 2 for 5–25% involvement; 3 for 26–49% involvement; 4 for 50–75% involvement; and 5 for > 75% involvement. Three types of CT manifestations (i.e., ground-glass opacity, paving stone sign, and consolidation) were assigned different weights. If a lung lobe showed a paving stone sign, the baseline CT score was increased by 1; if it showed consolidation (with or without a paving stone sign), the baseline CT score was increased by 2. The total CT score was the sum of the scores for each of the five lung lobes, ranging from 0 to 35.

### Statistical analysis

Statistical analysis was conducted using GraphPad Prism 8.0. Continuous variables with normal distribution are expressed as the mean ± standard deviation, and those with a non-normal distribution are defined as the median (interquartile range). An unpaired *t*-test, nonparametric *t*-test, or Mann–Whitney U test was used to compare the differences between the two groups. One-way analysis of variance was used to compare multiple continuous variables. Categorical variables were expressed as percentages and compared between two groups by chi-square test, continuity correction test, or Fisher’s exact test. Kaplan–Meier analysis was used to evaluate the effect of lavage times and time on hospitalization time, and the log-rank test was used to compare the results. Multivariate logistic regression analysis of laboratory tests, BS, CT score, SOFA score and APACHE II score was used to screen independent risk factors for SMPP, and a nomogram model was established to predict SMPP risk using R 4.2.2 software. The bootstrap method was used to perform internal validation of the model, and a calibration curve and receiver operator characteristic (ROC) curve were drawn to evaluate the accuracy and robustness of the model. The Hosmer–Lemeshow test was used to assess the model’s goodness of fit. P-values < 0.05 indicated statistical significance.

## Results

### Patient selection

**Fig. 1:**
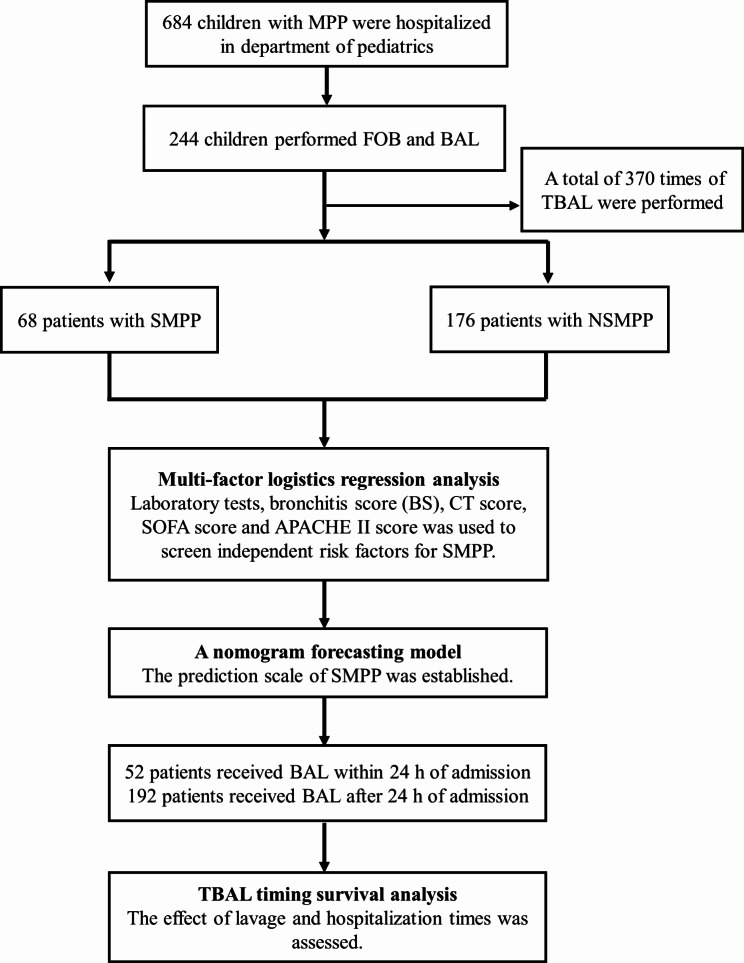
Research flow chart *MPP: Mycoplasma pneumoniae pneumonia, SMPP: Severe mycoplasma pneumoniae pneumonia, NSMPP: non-severe mycoplasma pneumoniae pneumonia, TBAL: Therapeutic bronchoalveolar lavage, FOB: Fiberoptic bronchoscopy, SOFA: Sequential organ failure assessment, CT: Computed tomography, APACHE: Acute Physiology and Chronic Health Evaluation

From January 2019 to December 2021, 684 children with MPP were admitted to the Department of Pediatrics of the First Affiliated Hospital of Xinxiang Medical University, and 244 patients underwent TBAL treatment and completed the study, including 68 with SMPP and 176 with NSMPP. According to the FOB time, 52 and 192 patients were classified into the early and late groups, respectively. A total of 370 FOB and BAL treatments were performed in the enrolled children (Fig. 1).

### Comparison of clinical features between the SMPP and NSMPP groups

Compared to the NSMPP group, the SMPP group had a significantly younger age (*P* = 0.00), less fever, cough time, and medical history (*P* < 0.0001). The SMPP group also had a higher incidence of dyspnea than the NSMPP group (*P* < 0.0001) but no significant difference in the incidence of extrapulmonary complications (*P* = 0.717). The GMPP group had more pleural effusion than the SMPP group (*P* = 0.0004). The SMPP group had a longer hospitalization time, higher APACHE II score, and SOFA score than the NSMPP group (*P* < 0.05). In contrast, the NSMPP group had a higher probability of RMPP (*P* < 0.0001). Regarding CT score, except for the right middle lobe and right lower lobe, there was no significant difference in total score and each lobe score between the SMPP and NSMPP groups (*P* > 0.05). Regarding BS, the SMPP group had increased mucosal pallor compared to the NSMPP group (*P* = 0.028). Additionally, the SMPP group had higher LDH, immunoglobulin abnormality rate, and NK cell abnormality rate than the NSMPP group, however, the MP-DNA sequence is lower (*P* < 0.0001) (Table 1).

**Table 1:** Comparison of clinical features between the SMPP and NSMPP groups [*n* (*%*), $\bar x \pm s$]

	Index	Total	SMPP (n = 68)	NSMPP(n = 176)	*Z/χ2*	*P*
	Sex, male	132 (54.1)	32 (47.06)	100 (56.82)	1.881	0.1701
	Age, y	5.25(1.75,7)	0.875(0.2604,6.25)	6(3.31,8)	5.922	0.00
Clinical data	Fever, d	7(3,10)	3(1,7)	9(6,13)	7.129	0.00
	Cough, d	6(3,10.25	3(1.25,7)	7(4,13)	4.689	0.00
	Dyspnea, n	47 (19.26)	38 (55.88)	9 (5.11)	81.2905	< 0.0001
	Extrapulmonary complications, n	123 (50.41)	37 (54.41)	86 (48.86)	0.1312	0.717
	Pleural effusion, n	68 (27.87)	8 (11.76)	60 (34.09)	12.162	0.0004
	Prehospital history, d	7(5,12.75	5(2,8.75)	9(6,14)	5.179	0.00
	Hospitalization time, d	13(10,17)	14(10,26)	12(10,15)	3.071	0.002
	APACHE II	3(0,7.75)	11(8,13.75)	2(0,3)	11.511	0.00
	SOFA	2(0,4)	5(4,7)	1(0,2)	10.251	0.00
	Times of BAL	1(1,2)	1.5(1,2)	1(1,1)	4.469	0.00
	Plastic bronchitis, n	54 (22.13)	10 (14.71)	44 (25)	3.306	0.069
	RMPP, n	105 (43.03)	15 (22.06)	90 (51.14)	16.917	< 0.0001
CT score	Total	10(8,14)	11(8,14)	10(8,14)	0.496	0.620
	Right upper lobe	2(0,4)	3(1.25,4)	1(0,4)	0.496	0.620
	Right middle lobe	2(0,4)	3(1,4)	1(0,4)	3.561	0.000
	Right lower lobe	2(1,4)	2(1,4)	2(0.25,4)	2.818	0.005
	Left upper lobe	1(0,2)	1(0,2)	1(0,3)	0.166	0.868
	Left lower lobe	2(0,4)	2(0,3)	2(0,5)	0.321	0.748
BS	Total	10(9,12)	10(9,12)	10(9,12)9	0.863	0.388
	Secretion volume	5(4,6)	5(4,6)	5(4,6)	0.818	0.413
	Secretion color	3(2,3)	3(2,3)	3(2,3)	0.500	0.617
	Mucosal edema	2(1,2)	2(1,2)	2(1,2)	0.416	0.677
	Mucosal eminence	0(0,0)	0(0,0.75)	0(0,0)	1.718	0.086
	Mucosal erythema	1(1,1)	1(0,1)	1(1,1)	0.489	0.625
	Mucosal pallor	0(0,1)	0(0,1)	0(0,1)	2.196	0.028
Laboratory index	MP-DNA, copies/ml	128,000(1500,32900000)	2365(551,16800)	5,965,000(9655,70175)	6.210	0.00
	WBC, ×10^9^/L	9(6.51,12)	9(6,13)	9(7,12)	0.055	0.956
	CRP, mg/L	21.15(4.8,49.1)	14(4.25,59.25)	23(5.25,45)	0.213	0.831
	PCT, ng/ml	0.29(0.09,0.78)	0(0,1)	0(0,1)	0.665	0.506
	LDH, U/L	627.5(400.75,883.5)	730(543,1072)	566(381.5,875)	2.151	0.032
	IL-6, mg/L	25.54(10.87,82.7)	26(12.25,0.91)	28(10.25,75.5)	0.783	0.434
	DD	2.1(1,5.3)	2(1,5)	2(1,5)	0.114	0.909
	Immunoglobulin abnormality, n	40 (16.39)	23 (33.82)	17 (9.66)	20.5029	< 0.0001
	Abnormal NK cell ratio, n	42 (17.21)	24 (35.29)	18 (10.23)	21.6277	< 0.0001

*SMPP: Severe mycoplasma pneumoniae pneumonia, BS: Bronchitis score, CT: Computed tomography, RMPP: Refractory mycoplasma pneumoniae pneumonia, PCT: Procalcitonin, IL-6: Interleukin-6, NK: Natural killer, SOFA: Sequential organ failure assessment, APACHE: Acute Physiology and Chronic Health Evaluation, CRP: C-reactive protein, WBC: White blood cells, LDH: Lactate dehydrogenase, DD: d-dimer

### Comparison of clinical data between early TBAL group (receiving TBAL within 24 h of admission) and late TBAL group(receiving TBAL after 24 h of admission)

Compared to the late TBAL group, the early TBAL treatment group had a shorter hospitalization time (*P* = 0.0022) and a more significant reduction of MP sequence number (*P* = 0.002). However, there were no significant differences in the other clinical manifestations between the two groups (*P* > 0.05) (Table 2).

**Table 2:** Comparison of early and late TBAL group *[n (%), M (IQR)]*

Index	Early (n = 52)	Late (n = 192)	*Z/χ2*	*P*
Fever, d	7(3.25,12.75)	6(3,10)	0.169	0.866
Cough, d	7(3,12.75)	7(3,10)	0.419	0.675
Extrapulmonary complications, n (%)	28 (53.85)	95 (49.48)	0.312	0.576
Pleural effusion, n (%)	15 (28.85)	53 (27.60)	0.482	0.487
Hospitalization time, d	10(9,14)	13(10,17.75)	3.043	0.002
SMPP, n (%)	16 (30.77)	52 (27.08)	0.277	0.599
RMPP, n (%)	24 (46.15)	81 (42.19)	0.2626	0.608
APACHE II change	3(0,7)	3(0,8)	0.284	0.776
SOFA	2(0,3.75)	2(0,4)	0.061	0.951

* SMPP: Severe mycoplasma pneumoniae pneumonia, RMPP: Refractory mycoplasma pneumoniae pneumonia, APACHE: Acute Physiology and Chronic Health Evaluation, SOFA: Sequential organ failure assessment

### Multivariate logistic regression analysis

Taking SMPP as the dependent variable (assignment: NSMPP = 0, SMPP = 1) and taking the significant and infection-related indicators in the univariate analysis as independent variables for multivariate logistic regression analysis, the results showed that the left upper lobe CT score (odds ratio [OR] = 0.499, 95% confidence interval [CI]: 0.28–0.892), SOFA score (OR = 1.913, 95% CI: 1.295–2.826), APACHE II score (OR = 2.641, 95% CI: 1.812–3.848), BS score (OR = 1.833, 95% CI: 1.248–2.692), and CRP (OR = 0.98, 95% CI: 0.963–0.997) were independent risk factors for SMPP (*P* < 0.05) (see Table 3).

**Table 3:** Multivariate logistic regression analysis results of SMPP

Variables	B	SE	waldχ2	P	OR	95%CI:
CT score of the left upper lobe	−0.694	0.296	5.506	0.019	0.499	0.28–0.892
SOFA	0.648	0.199	10.605	0.001	1.913	1.295–2.826
APACHE II	0.971	0.192	25.549	< 0.001	2.641	1.812–3.848
Bronchitis score	0.606	0.196	9.544	0.002	1.833	1.248–2.692
CRP	−0.02	0.009	5.436	0.02	0.98	0.963–0.997
Constant	−13.032	3.059	18.147	< 0.001	0.000	

*CT: Computed tomography, SOFA: Sequential organ failure assessment, APACHE: Acute Physiology and Chronic Health Evaluation, CRP: C-reactive protein

### Establishment and validation of the SMPP nomogram

**Fig. 2:**
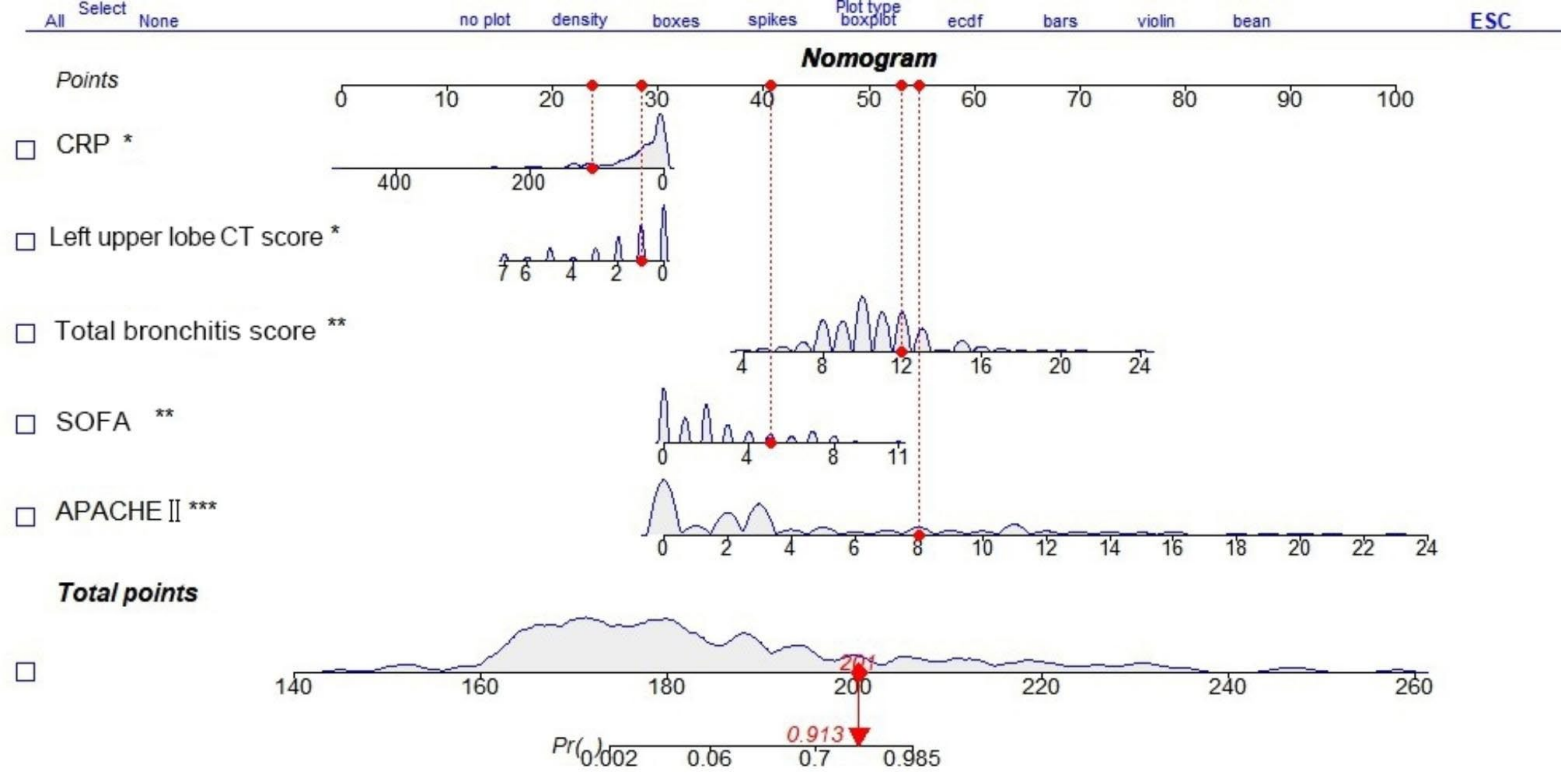
**SMPP nomogram prediction model**. The different values of each variable included in the nomogram have corresponding scores perpendicular to the scoring scale at the top; the sum of the scores for each item is the total score, which corresponds to the probability of death on the prediction probability line at the bottom. For example, for one child with MPP, a CRP of 110 mg/L corresponds to a score of 24, a left upper lobe CT score of 1 point corresponds to a score of 28.5, a bronchitis score of 12 points corresponds to a score of 53, a SOFA score of 5 points corresponds to a score of 41, and an APACHE II score of 8 points corresponds to a score of 54.5, will result in a corresponding total score of 24 + 28.5 + 53 + 41 + 54.5 = 201, with a probability of developing SMPP of 0.913, meaning that this child will have a very high risk of developing SMPP and may need closer monitoring and treatment * SOFA: Sequential organ failure assessment, CT: Computed tomography, APACHE: Acute Physiology and Chronic Health Evaluation, CRP: C-reactive protein

The multivariate logistic regression analysis results established a model with left upper lobe CT score, SOFA score, APACHE II, BS score, and CRP variables. The scores of each factor were summed to obtain the total score, which was converted to the predicted probability. A nomogram was constructed to predict SMPP (see Fig. 2).

### ROC curve and a calibration curve of the SMPP prediction model

**Fig. 3:**
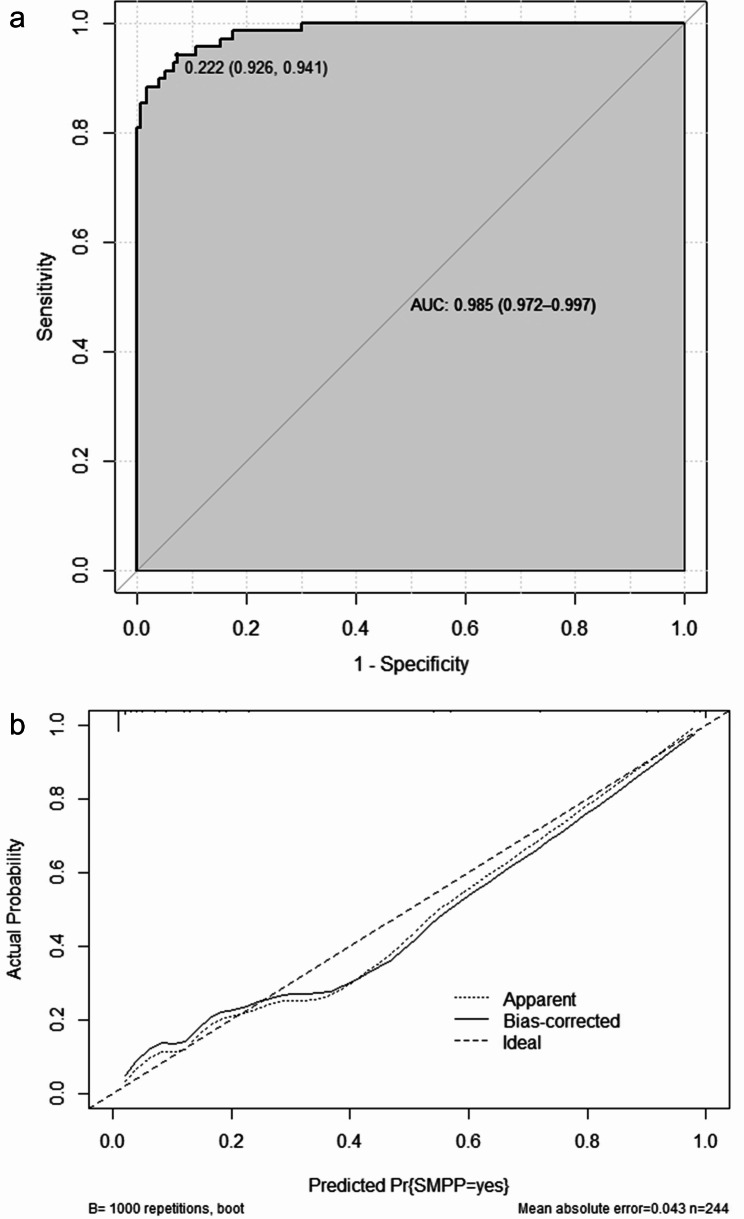
**SMPP prediction model ROC curve**. (**a**) The ROC curve shows an AUC of 0.985, with a 95% CI of 0.972–0.997, a 0.222 cut-off value, and sensitivity and specificity of 0.926 and 0.941, respectively. (**b**) The Hosmer–Lemeshow goodness-of-fit test results show that the nomogram model predicted SMPP risk with a good consistency (χ2 = 2.127, P = 0.977) * AUC: Area under the curve, SMPP: Severe mycoplasma pneumoniae pneumonia

The SMPP prediction model ROC curve showed an AUC of 0.985 (95% CI: 0.972–0.997), a cut-off value of 0.222, and a sensitivity and specificity of 0.926 and 0.941, respectively (Fig. 3). The Hosmer–Lemeshow goodness-of-fit test results showed that the nomogram model predicted SMPP risk with a good consistency (χ2 = 2.127, P = 0.977).

### Survival analysis of TBAL treatment timing on length of hospital stay

**Fig. 4:**
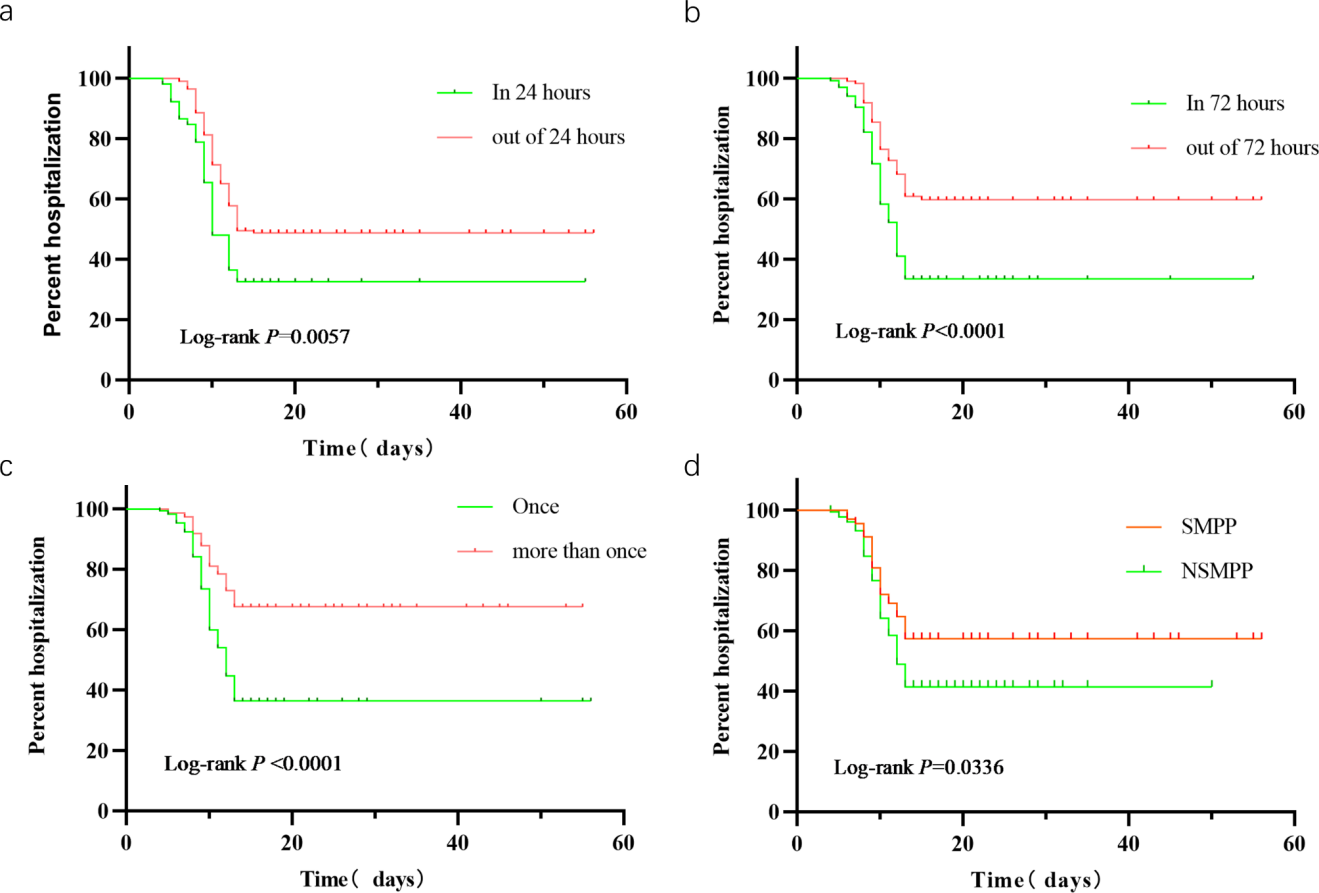
**Survival analysis of TBAL timing on the length of hospital stay**. (**a**). In 24 h vs. out of 24 h:log-rank *P* = 0.0057, 95% CI: 0.5229–1.132. (**b**). In 72 h vs. out of 72 h:log-rank *P* < 0.0001, 95% CI: 1.522–3.123. (**c**). Once vs. more than once:log-rank *P* < 0.0001, 95% CI: 1.698–3.472. (**d**). SMPP vs. NSMPP:log-rank **P** = 0.033, 95% CI: 0.4529–0.9535 * SMPP: Severe mycoplasma pneumoniae pneumonia, NSMPP: non-severe mycoplasma pneumoniae pneumonia

We next performed a survival analysis on the impact of the number and timing of TBAL on hospitalization time, taking the median discharge time of 13 days as the survival time and discharge within 13 days as positive. The results showed that patients who underwent TBAL within 24 h of admission had significantly shorter hospitalization time than those who underwent TBAL after 24 h and had a higher discharge rate within 13 days than the latter (log-rank *P* = 0.0057, 95% CI: 0.5229–1.132; Fig. 4a). Similarly, patients who underwent TBAL within 72 h had a significantly shorter hospitalization time than those who underwent TBAL after 72 h, and had a higher discharge rate within 13 days than the latter (log-rank *P* < 0.0001, 95% CI: 1.522–3.123; Fig. 4b). Patients with TBAL once had a higher discharge rate within 13 days than those with more than two TBALs (log-rank *P* < 0.0001, 95% CI: 1.698–3.472; Fig. 4c). Figure 4d shows that the patients with SMPP had a longer hospitalization time than those with NSMPP (log-rank *P* = 0.033, 95% CI: 0.4529–0.9535).

## Discussion

### Differences in clinical manifestations between SMPP and NSMPP

SMPP can cause life-threatening complications and mortality in children [15]. Our results showed that the SMPP group had a shorter history of pre-hospitalization, fever, and cough time than the NSMPP group but more severe clinical manifestations such as dyspnea. These findings are similar to those reported by Izumikawa [3] and Lee [4]. However, our patients showed a lower incidence of pleural effusion than those in Lee’s study. Our study also found that patients with SMPP had fewer RMPP cases than those with NSMPP, which may be a novel finding. In this study, patients with SMPP had higher rates of immunoglobulin and NK cell abnormalities than those with NSMPP, indicative of humoral and cellular immunity dysfunction, suggesting that mycoplasma pneumoniae infection can cause host innate immunity and adaptive immunity disorders, further aggravating respiratory system damage [16]. However, the mechanism underlying the MP damage to the immune system remains to be further studied.

### Establishment of nomogram forecasting model of SMPP

Our results also showed that the left upper lobe CT score, SOFA score, APACHE II score, BS score, and CRP level were independently associated with SMPP. These variables reflect different aspects of the pathophysiology of SMPP, such as the pulmonary inflammatory response, organ dysfunction, and systemic physiological response. Based on these risk factors, we constructed a nomogram model to predict the probability of SMPP in children with MPP. A nomogram model is a graphical representation of a prediction model that can provide individualized risk estimates by summing the points assigned to each variable, which may have value as biomarkers for SMPP [17]. Our nomogram model can be easily applied to clinical practice using a scoring system based on five variables.

The SOFA score, APACHE II score, and CRP are important condition assessment systems to evaluate the severity of patient’s condition and prognosis in the ICU [18] and provide a basis for clinical decision-making. The most recent study [19] found that the APACHE II score on day 3 of ICU admission was the best predictor, while another found that SOFA score was the best to predict in-hospital mortality in patients with severe surgical lung infections [18]. Therefore, for patients with SMPP, we recommend early risk management, performance, and early outcome prediction.

CT and other imaging scores are widely used in assessing lobar and interstitial pneumonia [14], which are conducive to quantitatively assessing pulmonary inflammation. The higher the CT score, the greater the chance of lung compactness or atelectasis. Luo et al. [20] proposed that bronchoscopy and BAL should be performed as soon as possible after the diagnosis of atelectasis to reduce the risk of multiple bronchoscopy and improve the therapeutic effect of BAL. We showed that patients with high CT scores in the upper left lobe were more likely to develop SMPP, which may be related to the high anatomical position and the smaller and more numerous bronchial subsegment openings, which can affect the spread and resolution of lobar pneumonia [21]. BS is a quantitative assessment of bronchial inflammation by bronchoscopy [9], which can directly evaluate bronchial wall destruction and endobronchial sputum blockage. Many children with MP-induced atelectasis or consolidation had a large amount of secretion in the bronchus, and may only display endometrial pale or erythema. Therefore, BS combined with CT score was more comprehensive than either alone in assessing the changes of pulmonary inflammation. Early FOB and BAL can rapidly detect pathogens and identify drug resistance genes through sequencing, which is conducive to the precise treatment of severe infections [22].

We develop a nomogram model to predict SMPP in children and to evaluate using multiple aspects such as imaging, bronchoscopy, condition scoring, and inflammatory indicators. Zhang’s RMPP plastic bronchitis nomogram prediction model is mainly based on clinical manifestations such as fever, extrapulmonary complications, pleural effusion, cough duration, and lactate dehydrogenase [23]. Zhao et al. mainly used laboratory indicators such as peak body temperature, neutrophil ratio, platelet count, interleukin-6, lactate dehydrogenase, and atelectasis [24]. Both studies are less detailed and comprehensive regarding disease assessment, and rarely nomogram study on SMPP prediction has been conducted.

### TBAL timing recommendations based on nomogram prediction models

Our previous study showed that early BAL could reduce hospitalization and ICU time [9]. Previous studies have shown that BAL can effectively treat children with refractory MPP or MPP complicated with atelectasis and reduce cytokines [8, 10, 25]. However, there are no clear guidelines on when to perform TBAL for children with MPP [26], whether early TBAL is more effective than late TBAL remains unknown.

We used Kaplan–Meier analysis to evaluate the impact of lavage times and duration on hospitalization time. Our findings indicate that early TBAL treatment was beneficial in reducing the length of hospitalization, which aligns with the study conducted by Lu et al. [10]. This may be because early TBAL can detect pathogens in the bronchoalveolar lavage fluid at an early stage and allow for the adjustment of sensitive antibiotics, thereby facilitating the clearance of pathogens [27].At the same time, bronchitis score was an independent predictor in the nomogram of this study, which means that early intervention TBAL can be used to evaluate and prevent MPP from developing into SMPP or reduce the severity of SMPP.

However, we also found that multiple TBAL treatments are not necessarily beneficial, which may be related to the severity of the condition. SMPP patients already experience significant lung inflammation and damage, and multiple TBAL may increase the risk of complications such as bleeding, infection, or pneumothorax [28], which may necessitate additional interventions and prolong the hospital stay. Wang et al. [8] also showed that BAL treatment increased the secondary infection rate and hospitalization time compared to no BAL. Therefore, after predicting SMPP using the nomogram model, the subsequent TBAL should be conducted according to the patient’s condition [29, 30].

### Limitations

Our study has some limitations that should be acknowledged. First, this was a retrospective study with a relatively small sample size from a single center. Second, we did not use an independent cohort or prospective data to externally validate our nomogram model. Therefore, further studies are needed to validate our model in multicenter, large samples, and diverse environments and populations.

## Conclusion

In summary, we established a nomogram model based on the left upper lobe CT score, SOFA score, APACHE II score, BS, and CRP level to predict the risk of SMPP in children with good accuracy and robustness. Additionally, early FOB and BAL treatment helped evaluate SMPP children’s condition and optimize TBALtiming.

## Data Availability

All data generated or analyzed during this study are included in this article.

## Abbreviations

APACHE: Acute Physiology and Chronic Health Evaluation
BAL: Bronchoalveolar lavage
BS: Bronchitis score
CI: Confidence interval
CRP: C-reactive protein
CT: Computed tomography
FOB: Fiberoptic bronchoscopy
IL-6: Interleukin-6
LDH: Lactate dehydrogenase
MP: Mycoplasma pneumoniae
MPP: Mycoplasma pneumoniae pneumonia
NK: Natural killer
NSMPP: Non-severe mycoplasma pneumoniae pneumonia
OR: Odds ratio
PCT: Procalcitonin
RMPP: Refractory mycoplasma pneumoniae pneumonia
ROC: Receiver operator characteristic
SMPP: Severe mycoplasma pneumoniae pneumonia
SOFA: Sequential organ failure assessment
TBAL: Therapeutic bronchoalveolar lavage
WBC: White blood cells
